# Placenta Previa

**Published:** 1881-01

**Authors:** J. P. Thomas


### PLACENTA PREVIA.

By J. P. THOMAS, M.D.

While few subjects in the whole domain of obstetrics have (during the past two centuries) given rise to more discussion than placental presentations, it is only within the last fifty years that any real advance in the management of this freak of nature has been made. The ancients believed this abnormal position of the placenta to be the result of accident whereby it was detached from its proper site, to this being superadded the force of gravity. Portal is generally credited with the discovery that this position of the "after-burden," as he called it, was an original insertion of the placenta over or adjacent to the os internum. Cazeaux ascribes the discovery to Gifford, and, without mentioning Portal, credits Levret with having first directed attention to it, and demonstrated its frequency and danger, as well as pointing out the proper mode of detecting its existence, and adds, "The insertion of the placenta over the os uteri has been considered since the days of Levret as an inevitable cause of hemorrhage during the last three months of gestation and during the course of parturition."

Though the causes of placenta previa have been variously stated by different authors, our actual knowledge of the subject amounts positively to nothing. We know but little more than that perhaps once in six or seven hundred cases the placenta is placed over or near the os uteri. Cazeaux thought that the uterine mucous membrane is perhaps less swollen and turgid than when conception takes place in the natural attachments of the placenta, and that therefore it offers less obstruction to the descent of the ovule to the lower part of the uterine cavity, etc. Tyler Smith held that the ovule does not become impregnated until it reaches the lower part of the uterus in the preplacental attachments. But the cause of this location of the placenta is as far from being understood now as it was two hundred years ago.

There are perhaps equally as many theories as to the source of the hemorrhage in placenta previa. I shall notice but one of these. Simpson, following Chapman, of Ampthill, England, suggested that the source of the flow is from the placental vessels, and not as is at present generally admitted, from the uterine surface. Hamilton, of Edinburgh, claimed that the hemorrhage proceeded from the separated portion of the placenta rather than from the ruptured uterine vessels. Simpson's earnest and able advocacy of the first theory, and the practice of detaching the placenta *en masse* which resulted, have been the cause of a most pernicious practice, namely, removing the placenta in all cases of placenta previa. This practice based as it is upon the false theory that the hemorrhage is placental and not uterine, has been the cause of the death of the child in many cases where by partial detachment of the placenta and immediate version or forceps-delivery both child and mother might have been saved. A mere glance at the anatomical distribution of the placental arteries, together with a study of its physiology, should, it seems to me, convince any one of the fallacy of this theory. Yet, because in so many of his cases the hemorrhage ceased on the removal of the plecenta, Simpson was, it seems, satisfied of its correctness. Tyler Smith, in dissenting from Simpson's theory, says, "Detachment of the placenta causes irritation, which excites the uterus generally, and the muscular structure at the site of the placenta especially, to contraction, and in this way the hemorrhage is prevented or arrested." Certain authors have erroneously declared that uterine contractions actually increase the flow of blood. Aside from the fact that contractions still further detach the placenta, there is no reason why they should not aid in arresting hemorrhage, as they clearly do in post-partum flooding in natural labor. Speaking from individual observation, I believe that uterine contractions perform the same office in arresting hemorrhage in partial or complete placenta previa that they do in flooding after natural labor, Barnes' zonular theory to the contrary notwithstanding, and that the apparent increase of the hemorrhage is, as stated by Playfair, but the expulsion of the blood already accumulated. Yet that Simpson's practice of detaching and removing the placenta is in some cases most excellent practice, experience teaches me. This opinion is based upon an experience of three cases, one of which I will briefly report. In this a centrally inserted placenta was detached and removed from necessity.

Mrs. W., aged thirty-eight, had six natural labors. In three of her pregnancies she had during the eighth month slight hemorrhages—the last, however, sufficiently profuse to demand a vaginal examination. I had difficulty in introducing a single finger, yet the diagnosis of central implantation was satisfactorily made out. The situation, with its attendant dangers, was fully explained to the husband, and premature delivery advised. But neither his entreaties nor my advice won her consent. I therefore enjoined perfect quiet in the horizontal position, and left instructions to call me immediately should hemorrhage recur. This advice was not followed. The patient continued to attend to her household duties, and, as I afterward learned, had attacks of hemorrhage every five or six days which, however, ceased on lying down. Two weeks later I was sent for in great haste to find her in active labor, the os dilating rapidly, the placenta covering the cervix except a space large enough to permit the finger to touch the bag of waters and detect a vertex presentation. The patient, however was so alarmingly prostrated from loss of blood that turning at that time was out of the question. I did not then, as now, have Barnes' invaluable dilators, and, fearing the tampon would but complicate the case by substituting a concealed for an opén hemorrhage, I administered a full dose of ergot, and detached and after ligating the cord, removed the placenta. I hoped that the bag of waters would take the place of the tampon and acting as a plug from within arrest the hemorrhage at least long enough to enable the patient to rally sufficiently to stand the operation of turning if not that of rupturing the membranes and delivering by forceps. But in detaching the placenta I unintentionally ruptured the membranes, whereupon the head came down, the vertex engaged the os, and all hamorrhage immediately ceased. This fortunate result was not attained because the source of the hemorrhage was placental, nor by the contractions of the uterus alone, but appeared mainly due to the mere mechanical effect of the child's head, which, occupying the former site of the placenta, made such pressure upon the open mouths of the uterine veins that it served as a veritable plug. The contractions, stimulated by the ergot, were frequent, forcible, and somewhat prolonged, and must have assisted somewhat in arresting the hemorrhage, not only by closing the mouths of the vessels and hastening the descent of the head, but also by causing the bleeding surface to hug the head more closely. The pains were so strong and persistent and the head now so low in the pelvis that turning became impossible; and even had it not been, the patient after two hours of exemption from hemorrhage, but in severe labor, was too much exhausted for the procedure; so the forceps were applied and a dead child delivered. Considering the excessive loss of blood and the severity of the pains, which were allowed to continue for so long a time because of not having my forceps at hand, the mother made a good recovery.

The foregoing case will serve to show that Simpson's practice of removing the placenta and leaving the case to nature altogether loses sight of the safety of the child. I think there are cases of central placental presentation, where the os is dilating or dilatable, and the labor has set in at full term or sufficiently near it to insure the viability of the child, when the safest practice for both mother and child would be the detachment and removal of the placenta and prompt delivery either by version or the forceps, the choice being determined by the circumstances of the individual case. In two such instances I have resorted to this procedure with the result of saving both mother and child, when I believe any other course would have resulted fatally to the children if not to the mothers also. The pressure of the head upon the bleeding surface of the uterus where the entire placenta is removed is not, I think, as fully appreciated by authors as it should be; for certainly where contractions are active, and the head has descended well and is still advancing, this always acts as a plug and thus effects the arrest of the hemorrhage. May it not have been the most efficient cause in many cases reported by Dr. Simpson and others? The following case will, I think, go to prove that the child is not lost by simple waste of blood, but by asphyxia; while it will serve to show that the source of the hemorrhage is principally uterine, and not placental:

Several years ago I saw, in consultation with the late Dr. Porter, Mrs. M., about thirty years of age, a previously healthy woman, accustomed to work, the mother of four living children coming after natural and easy labors, these attended by a midwife only. I reached her bedside only in time to find her dying of hemorrhage caused by a centrally-implanted placenta, and she still undelivered. She gasped but three or four times after my arrival, and expired. She was flooding profusely when Dr. P. was called in, and was so much prostrated that he resorted to the tampon, using for the purpose a silk handkerchief, which, I may remark in passing, answers an excellent purpose where vaginal tampons are applicable, which, though contrary to high authority, I think are very few. The placenta still occupied an almost central position, with its margin only undetached, the os being sufficiently dilated to engage the head. With faint hopes of saving the child, as very feeble fetal movements could be felt, I performed cesarian section within twenty minutes of the death of the mother—perhaps less time—and a well-developed male child at full term, partially asphixiated, but soon restored, was extracted. A few years ago he was a robust boy of ten years.

From 1870 to 1875 I produced premature delivery in three cases—one in the eighth month, one in the first week of the ninth and one only two weeks before term—with the result of saving the life of the mothers, but with the loss of two of the children. The seventh-month child was delivered after partial detachment of the placenta and podalic version. It was still-born. The eighth-month child was delivered after complete detachment of the placenta by immediate turning. Both child and mother lived and did well. The case in which labor was induced two weeks before the expiration of gestation was also delivered by turning, and nearly complete detachment of the placenta. The child lived only a few hours, and the mother had a tedious recovery, having an alarming attack of phlegmasia dolens.

In the past five years I have effected premature delivery in three cases after the seventh month, saving the lives of the mothers and one of the children. Though this so-called prophylactic treatment of placenta previa is indorsed by Barnes, Hewitt, Greenhalgh, and others, in

England, and by others of note as Thomas and Parvin in this country, I am persuaded that in some cases it is an unnecessary procedure and in others unjustifiable as to the safety of the child. I venture this opinion notwithstanding Thomas reports eleven cases in which the practice was resorted to solely as a prophylactic measure, with a loss of but five children and the death of one mother, and she from puerperal septicæmia, which might have occurred after natural labor. It is well to know that a miscarriage is generally more dangerous than labor at term, and that version at best is always attended with danger to the child from pressure on the cord. Consequently in cases where the hemorrhage is slight and infrequent, and the patient can be closely watched and controlled, and some one of the bromides combined with hyoscyamus be continuously given, they can generally be conducted safely to the end of gestation without impairing to any considerable extent, by loss of blood, the physical powers of the woman. The hypnotics presistently given seem to control even the insensible contractions of the uterus, to which the hemorrhage is certainly due in some cases. Two cases of marginal placental attachment under my care were thus conducted to full term, the hemorrhage in each case being slight and occuring at the menstrual period only. Both were safely delivered of living children after partial detachment of the placenta, and by podalic version, and within three weeks were well. In both cases the diagnosis of placental complication was clearly made out from the first hemorrhage.

Under such conditions then—that is where the flow is slight and infrequent, and having in view the dangers incident to miscarriages, and the undeveloped state of the fœtus, rendering its viability less certain—I ask, would I have been justifiable in either case in inducing premature delivery? In the history of Thomas' eleven cases this procedure was not only justifiable, but demanded in ten of them. But in his first case I think he should have temporized a little longer, at least. But as it is clearly impossible for the majority of general practitioners to give necessary attention in preplacental cases, and as it is an established fact that after the first occurrence of hemorrhage the woman is never free from danger until delivered, I am inclined to the opinion that in any case where the diagnosis is clear, the flow frequent and profuse, and the patient resides some distance from the medical attendant, premature delivery should be induced in the interest of the mother even before the period of viability of the child and without regard to its safety. But fortunately hemorrhage rarely occurs before this period. With Barnes' dilators there is but little risk of excessive hemorrhage, as they not only dilate the os sufficiently rapidly, but act so securely as a plug that in one of my cases the os was dilated by a gradual dilatation (three sizes being used), the placenta partially detached and turned back on itself, and the child delivered by version with but slight loss of blood, the uterus contracting promptly all the while.

There are cases in which nature proves herself competent to accomplish delivery with safety to both mother and child. In 1869 and 1870 two cases occurred in my practice which show what nature does sometimes accomplish, and how closely we imitate her when we induce premature delivery:

Mrs. C. was taken suddenly in labor, each pain being followed by a gush of blood. I reached her only in time to grasp the placenta as it emerged from the vulva. The labor was so rapid and the contractions so powerful, the child under size (the pregnancy being only seven months and two weeks advanced), it almost instantly followed the placenta enveloped in the membranes. The child lived a few hours only, but the mother made a rapid recovery.

Mrs. K.'s case was similar, except she was about eight and a half months advanced in pregnancy. Here both mother and child were saved.

In both the placenta was first expelled, the child and membranes followed almost instantly, and both made as good recoveries as after natural labor.

A case recently occurring in the practice of a confrere will still further illustrate how nature sometimes accomplishes safe delivery: Mrs. S., aged 26, mother of three children, was seen by Dr. L. He found her fearfully prostrated by hemorrhage, so much so that he thought the loss of a few ounces more would have resulted in death. The flooding had fortunately entirely ceased; the placenta lay compressed between the head and the os, one-third projecting into the vagina. He gave a large dose of fluid extract of ergot, and pressed the placenta back with his finger as the head advanced; no more hemorrhage occurred, labor proceeded, and a living, fully matured child was spontaneously delivered.

In 1878 Mrs. R. was taken in labor and simultaneously with free hemorrhage. Dr. ——, the family physician, was sent for, but being absent, Drs. —— and —— were called in. They found the patient extremely prostrated, though the hemorrhage had ceased. They detected a nearly centrally inserted placenta, and determined to endeavor to rally the woman by stimulants and bring her as quickly as possible to the point where version might be practiced. Just here the physician first sent for arrived, and declined to listen to the physicians in attendance proceeded at once to detach the placenta and turn the child. The shock proved too much for the already exhausted woman, and before the head escaped from the vulva she was dead. The child was alive, and still is, I believe.

Turning is the operation in placenta previa, and must continue to be with the general practitioner, and, in fact, with the great majority of the profession; first, because where the child has reached the period of viability, and is living, it offers the best chance, if performed before the mother has lost too much blood, for both mother and child; secondly, if the child is not viable or dead it is the quickest way to empty the womb of its contents, and thereby hasten contraction and save the mother; thirdly, it will be the operation most frequently resorted to, because comparatively few practitioners are supplied with the proper instruments, and when this is the case they seldom have them at hand, and they always have the best obstetrical instrument ever devised—the right arm and hand.

I have not alluded to the differential diagnosis of placenta previa because it is generally so easily made out by the touch and the mode and history of the development of the hemorrhage. I should perhaps add that I have never been able to detect the placental bruit in such cases as have occurred in my practice. In no case in which the os was sufficiently dilatable to admit either one or two fingers did the touch fail, whether the attachment was central or partial, to diagnose the case. If the insertion be marginal the os should be dilated sufficiently to admit two or more fingers, or if need be, the whole hand, in order to discover the placental site and establish the diagnosis.

During an obstetric practice of twenty-five years I have met with but thirteen cases of placenta previa. In these but one mother was lost, but seven of the thirteen children were either still-born or died—a mortality of over fifty per cent. Should further experience establish that premature delivery will lessen this fearful mortality, it should certainly always be performed whenever possible.

There is no emergency in the whole field of medicine which requires greater judgment or prompter action on the part of the physician than does placenta previa. The late Dr. A. K. Gardner, of New York, said: "Perhaps the greatest element requisite for an accoucher is decision; he should be able to recognize what is possible and what is impracticable, and early know what he can do and what he can not effect." He should always go armed with the best appliances for dilating the os uteri, for effecting speedy delivery, and arresting hemorrhage.

Since the above was read before the Society, the following case having occurred in my practice, I report it simply on account of its being so rare at so early a period of gestation:

C. R, mulatto, aged thirty, mother of three children, on October 30, 1880, was, after only two or three very severe pains, suddenly attacked with profuse uterine hemorrhage. The flooding was so alarming that I lost no time in examination, but immediately tamponed the vagina, with the effect of arresting the flow. No further hemorrhage occurring within two hours, the tampon was removed and the finger carried through the os, which, though rigid, readily allowed its introduction. I at once detected a presenting placenta, but in spite of the utmost gentleness of manipulation, there came a sudden gush of blood almost as profuse as the first. A portion of the placenta had been torn away by my finger sufficiently large to admit it into the uterine cavity, when the hemorrhage at once ceased. The ovum must have escaped in some of the numerous clots discharged. I gave ergot in full doses, but without further detachment of the placenta. The os was not dilated sufficiently to admit two fingers, and with but one finger I found the removal of the placenta impossible. The case was, therefore, left to nature, but closely watched. On the fourth day the placenta came away in two small sloughs. On the fifth day the patient had a severe chill lasting about three hours, followed by high fever, some tympanites, and the usual septic symptoms. One drachm of quinine given in doses of ten grains every three hours, together with four compound cathartic pills, arrested promptly and much to my surprise these dangerous symptoms. The patient stated that it had been two months and ten days since her last monthly show. She is now (November 10) convalescing.

